# Highly Sensitive Detection of Minimal Cardiac Ischemia using Positron Emission Tomography Imaging of Activated Platelets

**DOI:** 10.1038/srep38161

**Published:** 2016-12-02

**Authors:** Melanie Ziegler, Karen Alt, Brett M. Paterson, Peter Kanellakis, Alex Bobik, Paul S. Donnelly, Christoph E. Hagemeyer, Karlheinz Peter

**Affiliations:** 1 Atherothrombosis and Vascular Biology, Baker IDI Heart & Diabetes Institute, Melbourne, Australia; 2 Vascular Biotechnology, Baker IDI Heart & Diabetes Institute, Melbourne, Australia; 3 School of Chemistry and Bio21 Molecular Science and Biotechnology Institute, The University of Melbourne, Melbourne, Australia; 4 Vascular Biology & Atherosclerosis, Baker IDI Heart & Diabetes Institute, Melbourne, Australia; 5 Central Clinical School, Monash University, Melbourne, Australia; 6 RMIT University, Melbourne, Australia

## Abstract

A reliable method for the diagnosis of minimal cardiac ischemia would meet a strong demand for the sensitive diagnosis of coronary artery disease in cardiac stress testing and risk stratification in patients with chest pain but unremarkable ECGs and biomarkers. We hypothesized that platelets accumulate early on in ischemic myocardium and a newly developed technology of non-invasive molecular PET imaging of activated platelets can thus detect minimal degrees of myocardial ischemia. To induce different degrees of minimal cardiac ischemia, the left anterior descending artery (LAD) was ligated for 10, 20 or 60 min. Mice were injected with a newly generated scFv_anti-GPIIb/IIIa_-^64^CuMeCOSar radiotracer, composed of a single-chain antibody that only binds to activated integrin GPIIb/IIIa (α_IIb_β_III_) and thus to activated platelets, and a sarcophagine cage MeCOSar complexing the long half-life PET tracer copper-64. A single PET/CT scan was performed. Evans Blue/TTC staining to detect necrosis as well as classical serological biomarkers like Troponin I and heart-type fatty acid-binding protein (H-FABP) were negative, whereas PET imaging of activated platelets was able to detect small degrees of ischemia. Taken together, molecular PET imaging of activated platelets represents a unique and highly sensitive method to detect minimal cardiac ischemia.

Ischemic heart disease (IHD), a leading cause of death worldwide, is typically caused by coronary artery obstruction, most often as a consequence of atherosclerotic coronary artery disease (CAD)^1^. Currently, electrocardiogram (ECG) monitoring during exercise is widely used for an initial screening for the diagnosis and risk stratification of IHD^2^. To provide more reliable and accurate information on the location and extent of cardiac ischemia and thus to increase the diagnostic accuracy, additional imaging using nuclear medicine, echocardiographic or magnetic resonance stress testing is required. Clinically, SPECT, thallium-201 and technetium-99m, are routinely used for nuclear myocardial perfusion imaging^3^,^4^. PET imaging for stress testing has attracted major interest as it offers higher sensitivity (up to 90%), higher specificity (up to 89%), higher spatial and temporal resolution, and reliable attenuation and scatter correction compared to SPECT^5^,^6^. Despite these advanced imaging technologies, the majority of patients with CAD and thus at high risk of a myocardial infarction (MI) are not identified before they suffer from a MI, which may result in cardiac death or if survived in heart failure. To prevent MI, highly sensitive detection of non-symptomatic myocardial ischemia could be a way forward allowing prophylactic interventions with potentially enormous benefits for a large number of patients.

The sensitive detection of myocardial ischemia in patients presenting with chest pain often remains inconclusive as neither the ECG nor serological cardiac biomarkers such as troponins (Tn) are positive^7^,^8^. The general paradigm assumes that for these biomarkers to become positive necrosis in addition to ischemia has to occur^9^. In this diagnostic setting a sensitive detection method for ischemia would also provide a powerful tool for risk stratification of patients presenting with chest pain but unremarkable ECG and blood tests.

Platelets play a crucial role after transient myocardial ischemia and contribute importantly to reperfusion injury^10^. They are among the first wave of inflammatory cells to infiltrate the infarcted myocardium^11^ and platelet accumulation is strongly correlated with the location of ischemic and necrotic areas^12^. The major platelet integrin glycoprotein (GP)IIb/IIIa (α_IIb_/β_IIIa_; CD41/CD61) undergoes a conformational change upon platelet activation, which makes the activated conformation of GPIIb/IIIa a unique targeting epitope for the detection of activated platelets^13^. In addition, the fact that this receptor is highly abundant on platelets (60,000 to 80,000 receptors/platelet) and has exclusive expression on platelets in blood, make it an ideal target for molecular imaging without off-target binding^13^. We have previously generated specific single-chain antibodies (scFv) and have shown their unique suitability for imaging of activated platelets in thrombosis and inflammation using various imaging modalities such as ultrasound and MRI^12^,^14^,^15^. Most recently, we attached these scFvs to the sarcophagine bifunctional chelator MeCOSar thus generating an activated platelet targeted ^64^Cu radiotracer for PET^16^,^17^. Sarcophagines are macrobicyclic chelators that are particularly well suited for copper radiopharmaceutical applications because they form extremely stable complexes with ^64^Cu with fast complexation kinetics at room temperature, neutral pH and at low concentrations^18^,^19^,^20^,^21^.

In the present study, we hypothesized that PET/CT imaging of activated platelets could represent an attractive opportunity for the highly sensitive detection of cardiac ischemia. With the aim to determine the suitability of activated platelet-targeted PET imaging for the sensitive detection of cardiac ischemia, we induced different degrees of cardiac ischemia by temporary LAD ligation in mice. We generated the PET tracer, scFv_anti-GPIIb/IIIa_-^64^CuMeCOSar, which is both unique in its selective binding and targeting to activated GPIIb/IIIa and its complexing ability of radioactive copper. Using this targeted PET tracer, we provide proof of concept for a novel highly sensitive technology to detect and image cardiac ischemia.

## Results

### PET/CT imaging of activated platelets within the ischemic myocardium

**Figure 1:**
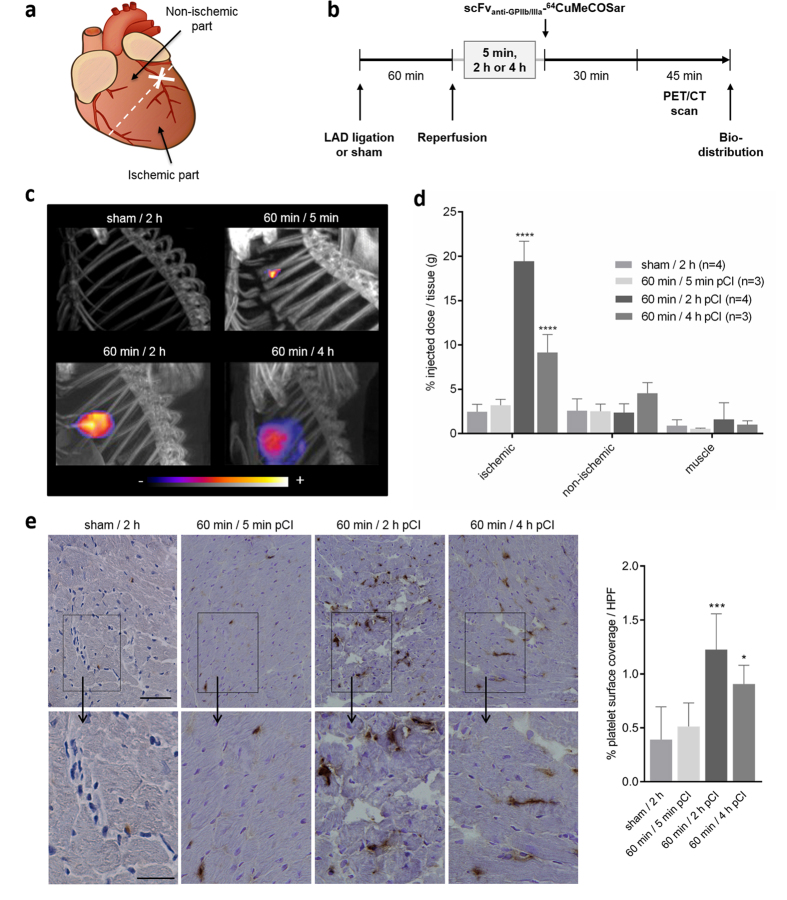
Time course of platelet accumulation in infarcted myocardium. Myocardial infarction was induced by 60 min LAD ligation. (**a**) For analysis, the heart was divided in an ischemic and a non-ischemic part. (**b**) Experimental protocol for PET/CT study. (**c**) Serial PET/CT images of activated platelets in the ischemic myocardium after injection of the scFv_anti-GPIIb/IIIa_-^64^CuMeCOSar radiotracer are shown. Comparison of representative maximum-intensity projection PET images at different time points (5 min, 2 and 4 h pCI). (**d**) Biodistribution analysis indicates a radioactive uptake of scFv_anti-GPIIb/IIIa_-^64^CuMeCOSar at different time points in the ischemic compared to the non-ischemic part of the myocardium and the muscle (n = 3–4, ****P < 0.0001, one-way ANOVA followed by Tukey’s multiple comparison test). Data are presented as mean ± SD. (**e**) 5 min, 2 h and 4 h pCI cardiac sections were immunostained for CD41. Representative images and quantitative analysis show increased platelet staining 2 and 4 h pCI (top scale bar: 100 μm, bottom scale bar: 50 μm, n = 4, *p < 0.05 and ***p < 0.001, one-way ANOVA followed by Tukey’s multiple comparison test, HPF: high power field).

We hypothesized that activated platelets accumulate in the ischemic myocardium. To determine the time course of platelet recruitment to the ischemic myocardium, transient myocardial ischemia of 60 min was induced and mice were injected intravenously with a single dose of the scFv_anti-GPIIb/IIIa_-^64^CuMeCOSar radiotracer 5 min, 2 or 4 h post cardiac ischemia (pCI) (Fig. 1a,b). Mice were then placed in a small animal PET/CT scanner. PET image acquisition of 30 min was performed 30 min post injection, directly followed by a CT scan. PET/CT scans showed a significant accumulation of the radiotracer in the myocardium 2 and 4 h pCI and no relevant uptake in the myocardium 5 min pCI or in the sham-operated animals (Fig. 1c, Supplementary Video S1). The biodistribution analysis confirmed a highly significant uptake of the radiotracer in the ischemic region of the myocardium 2 h pCI (19.46 ± 2.26% ID/g, *P* < 0.0001) and 4 h pCI (9.16 ± 2.03% ID/g, *P* < 0.0001) compared to 5 min pCI (3.2 ± 0.67% ID/g) and the sham-operated animals (2.46 ± 0.84% ID/g, Fig. 1d). In contrast, the non-ischemic regions of the heart and the muscle, as additional soft tissue control, showed only a background signal. As a further control, healthy hearts were investigated and no appreciable tracer accumulation could be detected (data not shown). Taken together, the highest scFv_anti-GPIIb/IIIa_-^64^CuMeCOSar uptake detecting activated platelets was present 2 h pCI.

### Accumulation of platelets within the ischemic myocardium

In addition to the PET/CT study we immunostained post ischemic myocardial sections with an anti-CD41 antibody to confirm the presence of platelets. Significant accumulation of activated platelets could be detected at 2 and 4 h after reperfusion but not after 5 min after reperfusion or in sham-operated animals (Fig. 1e). In accordance with the PET/CT and biodistribution data, accumulation of the anti-CD41 antibody reached its peak 2 h pCI, where platelets were mostly co-localized with leukocytes. This was followed by an observed reduction of the number of platelets within the ischemic myocardium at 4 h pCI.

In conclusion, activated platelets adhere after ischemia-reperfusion injury in the ischemic myocardium and reach a maximum accumulation after 2 h. Further PET/CT scans were performed 2 h pCI based on these results.

### Determination of ischemic and necrotic areas after different stages of myocardial ischemia

**Figure 2:**
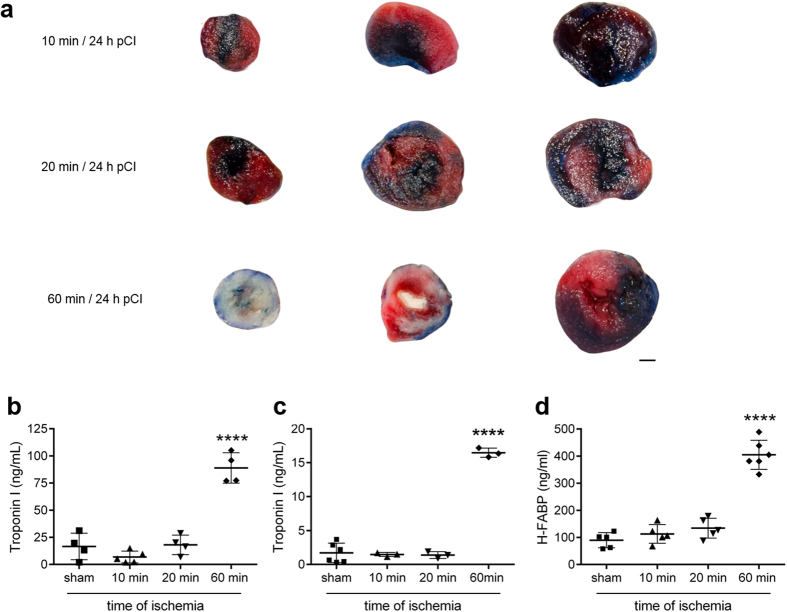
Diagnosis of myocardial ischemia by determination of ischemic area and biomarkers. Different stages of ischemia (10, 20 or 60 min of ischemia) were induced by transient LAD ligation. (**a**) Representative transverse cardiac sections show non-ischemic area (blue area), ischemic area (red area) and necrotic area (pale area) 24 h pCI (scale bar: 1 mm). (**b**–**d**) TnI and H-FABP serum levels were analysed 2 h and 24 h pCI. TnI concentration 2 h (**b**) and 24 h (**c**) pCI and (d) H-FABP level 2 h pCI (n = 3–6, ****P < 0.0001, one-way ANOVA followed by Tukey’s multiple comparison test). Data are presented as mean ± SD.

To estimate ischemic and necrotic areas, mice were exposed to three different episodes of cardiac ischemia (10, 20 and 60 min). The hearts were stained with Evans Blue/triphenyltetrazolium chloride (TTC) 24 h pCI (Fig. 2a). TTC is used to differentiate between metabolically active (turns red) and inactive tissue (stays pale). As expected, mice exposed to 60 min of ischemia showed abundant myocardial necrosis (pale area). In contrast, mice undergoing shorter periods of ischemia (10 and 20 min) did not show any necrotic, inactive areas in the ischemic myocardium (red area). Therefore, 10 and 20 min of ischemia are suitable to induce transient ischemia without necrosis and thus irreversible muscle damage.

### Troponin I levels in ischemic mice as a marker for myocardial necrosis

Serological biomarkers such us cardiac TnI and TnT are standard tests for the diagnosis of MI. Troponin proteins are well-known for their high sensitivity to myocardial necrosis and thought to be released into the circulation from dead or dying myocytes^22^,^23^.

To determine TnI levels at different stages of myocardial ischemia, mouse blood was collected 2 and 24 h pCI. Two hours pCI, the serum level of TnI in sham-operated animals showed a mean of 16.6 ± 12.2 ng/ml (Fig. 2b). There was a highly significant increase of TnI to a mean of 88.9 ± 14.0 ng/ml after 60 min of ischemia (*P* < 0.0001). In contrast, short time intervals of ischemia (10 and 20 min) showed baseline TnI levels of 6.8 ± 5.4 ng/ml and 18.0 ± 9.1 ng/ml, respectively. A similar effect could be detected 24 h pCI (Fig. 2c). Mice, which underwent ischemia for 10 and 20 min, showed baseline TnI levels. A highly significant increase of serum TnI was measured in mice with 60 min of ischemia (*P* < 0.0001).

### H-FABP levels in ischemic mice as a marker for myocardial ischemia

H-FABP is a small, cytosolic protein in the myocardium and has recently attracted major interest as a novel biomarker for myocardial ischemia^24^. We included this biomarker in our study to validate the occurrence of transient ischemia. Serum H-FABP levels 2 h pCI were measured using a mouse H-FABP ELISA. A baseline level of 89.6 ± 28.0 ng/ml was determined in sham-operated mice (Fig. 2d). A highly significant increase of H-FABP was detected in mice after 60 min of ischemia (404.9 ± 53.7 ng/ml, *P* < 0.0001). Short time intervals of ischemia (10 and 20 min) showed a non-significant increase of H-FABP to 112.8 ± 34.6 ng/ml and 134.1 ± 36.6 ng/ml, respectively. This non-significant increase of H-FABP indicates the presence of minimal cardiac ischemia.

### PET imaging of activated platelets to detect minimal myocardial ischemia

**Figure 3:**
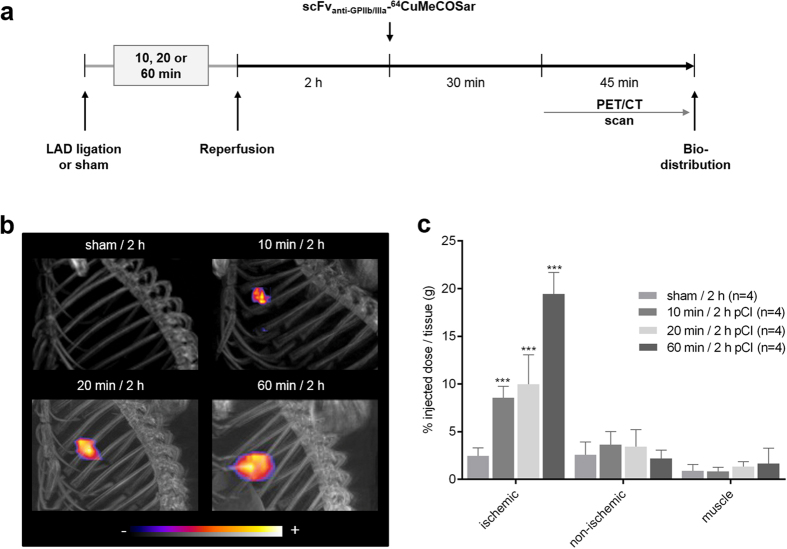
PET/CT imaging of activated platelets within the ischemic myocardium. (**a**) Experimental protocol for PET/CT study. (**b**) Comparison of representative maximum-intensity projection PET images after different stages of ischemia (10, 20 and 60 min). (**c**) Biodistribution analysis indicates a significantly higher radioactive uptake of scFv_anti-GPIIb/IIIa_-^64^CuMeCOSar in the ischemic compared to the non-ischemic part of the heart and the muscle (n = 4,***P < 0.001, one-way ANOVA followed by Tukey’s multiple comparison test). Data are presented as mean ± SD.

To evaluate the potential of PET imaging to detect minimal degrees of ischemia, mice underwent three different time periods of ischemia (10, 20 or 60 min) and were injected intravenously with a single dose of the scFv_anti-GPIIb/IIIa_-^64^CuMeCOSar radiotracer 2 h pCI (Fig. 3a). A PET/CT scan was performed 30 min post-injection. After only 10 min of ischemia a significant uptake of radiotracer in the ischemic myocardium was detected. The uptake increased in correlation to the duration of ischemia ending with the highest uptake after 60 min of ischemia. In comparison, no accumulation was seen in the sham-operated animals (Fig. 3b). In addition, the biodistribution analysis based on gamma-counter data set *ex vivo* confirmed a highly significant uptake of scFv_anti-GPIIb/IIIa_-^64^CuMeCOSar after all periods of myocardial ischemia (10 min: 8.58 ± 1.2% ID/g, *P* < 0.0001; 20 min: 9.98 ± 3.09% ID/g, *P* < 0.0001 and 60 min: 19.46 ± 2.26% ID/g, *P* < 0.0001) compared to the respective non-ischemic area (3.38 ± 1.91% ID/g; 3.5 ± 0.57% ID/g and 2.19 ± 0.89% ID/g), muscle (0.85 ± 0.44% ID/g; 1.01 ± 0.1% ID/g and 1.13 ± 1.29% ID/g) as well as sham-operated animals (2.24 ± 0.88% ID/g, Fig. 3c). Taken together, platelet-based PET imaging offers the possibility of early and reliable detection of minimal myocardial ischemia.

### Detection of platelets after different periods of myocardial ischemia using flow cytometry and immunohistochemistry

**Figure 4:**
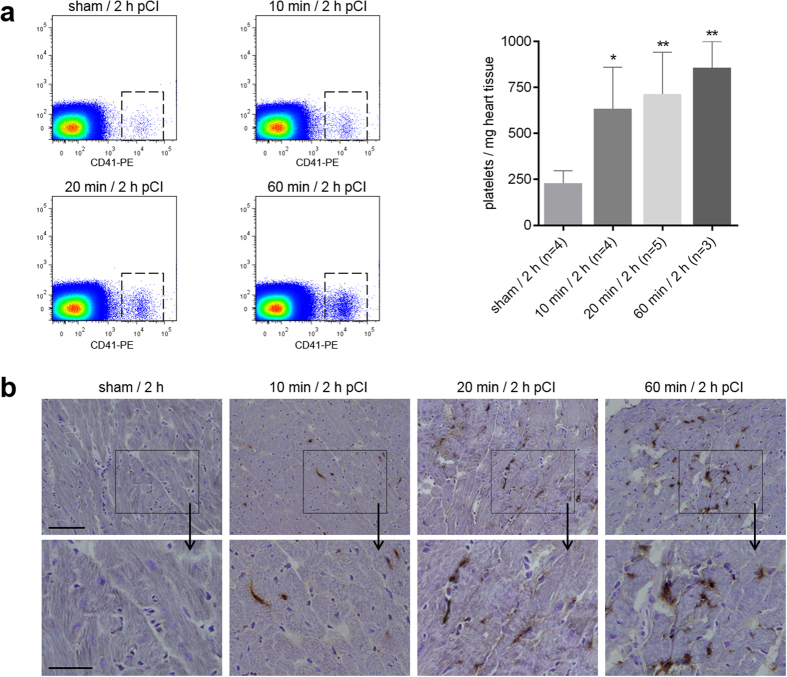
Detection of platelets within the ischemic myocardium. Mice underwent transient LAD ligation for 10, 20 or 60 min. (**a**) 2 h pCI, the ischemic part of the hearts was stained with anti-CD41-PE for flow cytometric analysis. Representative dot plots and quantitative analysis show a significant increase of CD41-positive events in heart samples that underwent 10, 20 and 60 min of ischemia compared to sham-operated animals (*P < 0.05, **P < 0.01, one-way ANOVA followed by Tukey’s multiple comparison test). Data are presented as mean ± SD. (**b**) Cardiac sections were immunostained for CD41. Representative images show platelet staining through all different stages of ischemia (top scale bar: 100 μm, bottom scale bar: 50 μm).

To confirm the PET/CT results, we determined the number of platelets in the ischemic myocardium by flow cytometry. Hearts were collected 2 h pCI, divided into ischemic and non-ischemic segments and enzymatically digested. A mean of 229.6 ± 112 platelets per mg heart tissue was found in the sham-operated animals (Fig. 4a). Similar numbers were found in the non-ischemic parts as well as the healthy heart (data not shown). After 10 min of ischemia the amount of platelets in the ischemic myocardium was significantly increased (618.5 ± 298 platelets per mg heart tissue, *P* < 0.05). A further increase was found in mice after 20 min of ischemia to 714.9 ± 227 platelets per mg heart tissue. Mice undergoing 60 min of ischemia resulted in 858.0 ± 142 platelets per mg heart tissue (*P* < 0.01, compared to the sham-operated animals).

These findings are supported by immunostaining of ischemic cardiac sections for the platelet-specific CD41 antigen. Recruitment of platelets into the ischemic areas could be found after all tested time intervals of myocardial ischemia (10, 20 or 60 min of ischemia) but not in sham-operated control tissue (Fig. 4b). The number of platelets found in the ischemic myocardium increased in correlation to the duration of ischemia, ending with the highest abundance of platelets after 60 min of ischemia.

## Discussion

The present study introduces a new non-invasive imaging technology for the detection of small degrees of cardiac ischemia. In current clinical practice, serological biomarkers play an important role in the diagnosis of cardiovascular diseases. In humans, troponins are specific and sensitive markers for myocardial damage and have become the biomarkers of choice to detect MI^22^,^25^. The question of whether cardiac biomarkers in general, or Tn in particular, are a suitable marker for reversible myocardial injury without necrosis has been debated for years and remains controversial^9^,^23^. In fact, troponin measurements are not used in routine clinical practice for the detection of ischemia in cardiac stress testing. Our data are consistent with the notion that either the extent of ischemia has to be substantial or necrosis has to occur for positive troponin readings. One of our major findings indicates that our novel PET imaging approach provides a higher sensitivity as compared to troponin and also H-FABP biomarker measurements.

Currently, 2–4% of patients with Acute Coronary Syndrome are discharged due to missed diagnosis and are mistakenly considered as not having CAD^26^,^27^. These are typically patients presenting to emergency departments with acute chest pain but negative ECG findings and unremarkable troponin tests who are stratified as low-risk patients^28^. However, this group represent a large proportion of CAD patients as their prevalence of cardiac events reaches up to 9.4% at long-term follow-up^29^. For this reason the American Heart Association (AHA) recommends an accelerated diagnostic protocol for these “low-risk patients” including a cardiac stress test to exclude ischemia^30^. A high sensitivity molecular PET imaging approach, such as the one described herein, could be highly valuable as a diagnostic tool in this often occurring clinical situation.

Our data shows that the scFv_anti-GPIIb/IIIa_-^64^CuMeCOSar radiotracer accumulation correlates well with the time of ischemia and is increasing with platelet abundance as detected by flow cytometry and immunostaining. These findings offer the new opportunity to use PET imaging of activated platelets for detection of minimal ischemia in the heart as well as potentially in other organs such us kidney and brain where current clinical practice is limited in its capability to detect ischemia.

Recently, cardiovascular MRI is increasingly used for the assessment of ventricular function and scarring using late gadolinium enhancement (LGE) and T1 mapping^31^,^32^. Contrast-enhanced MRI offers the possibility to image reversible and irreversible myocardial dysfunction and thus provides the opportunity to identify infarct size and hibernating myocardium^33^. For the assessment of stress-induced cardiac ischemia, stress wall motion and stress perfusion MRI using dobutamine or vasodilators are performed^33^,^34^,^35^. In addition, strain imaging in combination with dobutamine stress MRI has provided additional benefits in the evaluation of cardiac ischemia^36^,^37^. Overall, multiple studies over the last few years support the diagnostic superiority of either stress MRI or PET over the alternatives^36^,^38^. While MRI is a radiation free technique independent of cyclotron access, nuclear imaging such as PET has been seen advantageous in regards to its high sensitivity, reliability and accuracy towards the non-invasive assessment of myocardial ischemia and viability^39^,^40^. Prime examples are myocardial perfusion imaging with a very high diagnostic accuracy that is suitable to detect cardiac ischemia under stress^39^ as well as PET [^18^F]-FDG for myocardial viability assessment^41^, ultimately facilitating patient risk stratification^42^. The superior sensitivity in combination with suitable tracers such as the one presented in this study makes molecular PET highly attractive for the assessment of mild cardiac ischemia. The rapid evolution of hybrid PET/MR imaging promises to advance the field even further by providing detailed combined anatomic evaluation of coronary artery disease and functional alterations in cardiac function.

PET is an ideal imaging modality for this specific purpose and our approach based on ^64^Cu has a half-life of 12.7 h, which is favourable for clinical applicability compared to the short half-life of most common PET tracers (^13^NH_3_^82^, Rb, and ^15^O-labelled water). The longer half-life allows the radioisotope to be transported from the site of cyclotron generation to the clinic. Furthermore, radiochemical manipulations are more easily carried out with metal radioisotopes due to well-defined bioconjugation and coordination chemistry^43^,^44^. The use of a sarcophagine chelator to coordinate the ^64^Cu^II^ enables radiolabelling of the antibody conjugate at room temperature, generating constructs with high radiochemical purity and exceptional *in vivo* stability for imaging out to at least 48 h^18^,^19^,^20^,^21^. Our newly generated activated platelet-based PET imaging tool, due to the described advantages, has the potential for better diagnostic accuracy in cardiac stress testing.

For typical radionuclide stress tests two scans (under/after stress and at rest) have to be performed. Only the precise comparison between images under stress and at rest provides the necessary information to detect cardiac ischemia. In contrast, for the technology presented herein, patients will only need to undergo one single radiotracer injection as well as only one PET scan. As such, this is the first imaging technique that is able to visualize ischemia directly and not by comparisons to resting or non-ischemic areas. Cutting down the clinical standard procedure for cardiac stress testing and risk stratification by reducing both the radiotracer injection and the PET scan from currently two to only one is likely to reduce the financial burden for the health care system as well as the absolute exposure of radiation for patients.

## Conclusions

The described novel activated platelet-targeted PET imaging technology has the potential to detect and image small degrees of cardiac ischemia. Our data warrants further studies towards clinical translation of the described diagnostic technology. Platelet-targeted PET may be broadly applicable in various clinical diagnostic settings, such as highly sensitive cardiac stress testing as well as risk stratification for patients presenting with chest pain but unremarkable ECG or blood tests.

## Methods

### Mice

C57BL/6 mice were acquired from Jackson Laboratories and bred by Alfred Medical Research and Education Precinct (AMREP) Animal Services in Melbourne, VIC. All experimental work was performed in accordance with the Australian code for the care and use of animals for scientific purposes and approved by the AMREP Animal Ethics Committee (E/1402/2013/B).

### Myocardial ischemia and reperfusion

20–25 g weighing male, C57BL/6 mice were anaesthetized using a combination of ketamine HCl (100 mg/kg body weight (wt); Lyppard), xylazine HCl (5 mg/kg body wt; Lyppard) and atropine (1 mg/kg body wt; Pfizer) via intraperitoneal (i.p.) injection. Mice were orally intubated and ventilated throughout the procedure using a rodent ventilator (Model 687, Harvard Apparatus), with a tidal volume of 0.18 ml at 120 breaths/min. Mice underwent myocardial ischemia-inducing surgery by left anterior descending (LAD) coronary artery ligation as described previously^45^,^46^. To induce different stages of myocardial ischemia coronary occlusion was performed for 10, 20 and 60 min. Sham-operated mice underwent thoracotomy with a 7-0 suture placed around the LAD but not ligated.

### Evans Blue/TTC staining and immunohistochemistry

Mice were anaesthetized 24 h after surgery and the ischemic and infarcted area was estimated by Evans Blue/triphenyltetrazolium chloride (TTC) staining as described previously^47^,^48^. Briefly, the LAD was re-ligated with the same suture and 4% Evans Blue (AppliChem) was injected as a negative staining for perfused regions. The heart was then cut into 6 transverse slices and stained with 1% TTC (Sigma Aldrich) for 10 min. TTC added to metabolically active tissue turns healthy tissue red and the infarcted, necrotic myocardial tissue remains white.

Hearts were harvested, immediately fixed in 4% paraformaldehyde (PFA) and embedded in OCT Tissue Tec compound. Four defined heart sections for each mouse (5 μm) were immunostained using rat anti-mouse GPIIb (CD41) monoclonal antibody (1:200, Clone MWReg30, GeneTex), and a rat IgG1 isotype control antibody. The appropriate biotinylated secondary antibody (Vector Laboratories) was applied for 30 min at RT. Immunostaining of cardiac sections was performed with a streptavidin–biotin–immunoperoxidase method (Vectostatin ABC-Peroxidase and diaminobenzidine; Vector Laboratories) according to the manufacturer’s protocol. Samples were then imaged on an Olympus BX50F-3 microscope using a 200x magnification and five random images of each section were analysed using ImageJ 1.47 software. Results are expressed as % platelet surface coverage/high power field (HPF).

### Measuring of the biomarkers TnI and H-FABP

Blood was collected by cardiac puncture 2 h and 24 h pCI. Serum samples were diluted 1:6 in PBS. TnI serum levels were measured by the use of the Abbott Architect STAT High Sensitive Troponin-I (Abbott Diagnostics). Due to the high conservation between rodent and human TnI, this system is reliable in measuring rodent TnI levels. H-FABP serum concentration was measured 2 h pCI by a mouse-specific ELISA (Life Diagnostics) according to the manufacturer’s protocol. Serum samples were diluted 1:100 in PBS.

### Isolation of heart-infiltrating cells and flow cytometry

Two hours pCI the heart was removed, flushed with cold Krebs-Henseleit buffer (11 mM glucose, 1.2 mM MgSO_4_, 1.2 mM KH_2_PO_4_, 4.7 mM KCl, 118 mM NaCl, 25 mM NaHCO_3_, 1.25 mM CaCl_2_, pH 7.4) and divided in an ischemic and non-ischemic part (Fig. 1a). After mechanical disruption, the heart was digested for 2 h at 37 °C by an enzyme cocktail of 1.3 U Liberase DL Blendzyme (Roche) and 20 U DNase I (Ambion) in Tyrode-HEPES buffer (2.5 mmol/l HEPES, 150 mmol/l NaCl, 12 mmol/l NaHCO3, 2.5 mmol/l KCl, 1 mmol/l MgCl2, 5.5 mmol/l D-glucose, and 1 mg/ml BSA, pH 7.4). Cells were then passed through a 40 μm cell strainer, washed in 20 ml Tyrode-HEPES buffer, centrifuged at 300 *g* for 5 min and re-suspended in 400 μl Tyrode-HEPES buffer. Cells were then incubated with monoclonal anti-CD41-phycoerythrin (PE)-conjugated antibody for 30 min at RT. To determine the amount of CD41^+^ events per mg heart tissue PerCP Beads (BD Biosciences) were counted on a FACS Canto II (BD Biosciences) and used as reference. 1 μl PerCP Beads were added per 20 mg heart tissue and 2,000 PerCP Beads were counted on a BD FACS Canto II (BD Biosciences). FlowJo 7.6.5 were used for flow cytometry analysis.

### Production of the scFv_anti-GPIIb/IIIa_

ScFv_anti-GPIIb/IIIa_ was generated and produced as described previously^49^. Briefly, scFv DNA containing a C-terminal histidine protein purification tag was cloned in the pSecTag2a vector (Invitrogen) followed by transfection with polyethylenimine (Polyscience Inc.) into human kidney cells (HEK293F). After transfection, HEK cells were cultured for 7 days at 37 °C, with 5% CO_2_, shaking at 140 rpm. Supernatant was collected and purified with a nickel-based metal affinity chromatography column, Ni-NTA column (Qiagen), according to the manufacturer’s instructions. Purified scFv was dialysed against PBS at 4 °C overnight.

### ^64^Cu-production

No-carrier-added copper-64 (^64^Cu) was produced with the IBA Nirta target by the ^64^Ni(p, n)^64^Cu reaction. The target was produced by direct electroplating of highly enriched ^64^Ni (>99%, Isoflex USA) onto an Ag disk (24 mm diam × 1.0 mm thick disk). The plating cell was filled with ^64^Ni solution + NH_4_OH (total = 55 ml) and electroplating was carried out at 5.0 mA using a chopped saw tooth current for ~12–24 h to give an average 20–50 μm ^64^Ni thickness.

Targets were irradiated using an IBA 18/9 cyclotron with an incident beam of 14.9 Mev (18 MeV degraded by 0.5 mm aluminium foil). The irradiated disk was then loaded into an IBA Pinctada module and the ^64^Ni plating dissolved in recirculating 3 ml 9–12 M HCl at 70 °C using a peristaltic pump. Once dissolved, the solution was loaded onto an AG 1-X8 anion exchange cartridge for purification and the cartridge is washed with various concentrations of HCl to recover the ^64^Ni and elute impurities such as ^61^Co^64^. Cu was recovered in ~2 ml of water. Typical production yields average 30 mCi EOS for a 4 h irradiation at 35 μA with a ^64^Ni thickness of 25 μm (EOS 12 h post EOB).

### Production of MeCOSar-NHS-ester and scFv_anti-GPIIb/IIIa_-MeCOSar

MeCOSar-NHS-ester (where MeCOsar = 1-methyl-8-NHCO(CH_2_)_3_CO_2_H-sarcophagine; sarcophagine = 3, 6, 10, 13, 16, 19-hexaazabicyclo[6.6.6]-icosane = sar) as well as the conjugation of the MeCOSar to the scFv were prepared as described previously^17^,^50^. Briefly, the activated ester, (t-Boc)_4−5_MeCOSar-NHS-ester, was obtained by reacting (t-Boc)_4–5_MeCOSar with 1-ethyl-3-(3-(dimethylamino)-propyl)carbodiimide (EDC) and N-hydroxysuccinimide (NHS) in dimethylformamide (DMF), followed by purification by silica gel chromatography. The t-Boc groups were removed with trifluoroacetic acid, and MeCOSar-NHS-ester was isolated as the tris-trifluoroacetic acid tris-hydrate salt. Conjugation of the MeCOSar to the scFv was performed by incubating the scFv with MeCOSar-NHS-ester in PBS for 3 h at RT with shaking.

### Radiolabelling

ScFv_anti-GPIIb/IIIa_-MeCOSar (100 μg) was incubated with 25–35 MBq of ^64^Cu in PBS for 30 min at RT. A solution of 10 mM ethylenediaminetetraacetic acid (EDTA, 10 μl) was added and the reaction incubated for 5 min. All samples were washed twice with PBS using spin columns (Millipore, cut off 10,000 MWCO). Analysis/quality control was performed using thin layer chromatography (silica gel 60, F254; Merck) and 0.1 M citrate buffer (pH 5) as the mobile phase. Radiolabelled immunoconjugate (1.5 μl) was spotted at the origin, the strip was allowed to air-dry and was developed. The strip was cut into three pieces and the radioactivity in each section was counted using a gamma counter (Wizard single detector gamma-counter, Perkin Elmer).

### PET studies and post-mortem biodistribution

The animals were injected with scFv_anti-GPIIb/IIIa_-^64^CuMeCOSar (20 μg, 3–5 MBq), via lateral tail-vein injection. After 30 min incubation a PET scan was performed using NanoPET/CT *In Vivo* Preclinical Imager (Mediso, Hungary) with a 30 min PET acquisition time, and coincidence relation of 1–3. Image reconstruction was performed with the following parameters OSEM with SSRB 2D LOR, energy window, 400–600 keV; filter Ramlak cut off 1, number of iteration/subsets, 8/6. For the CT scans, an X-ray voltage of 45 kVp, an exposure time of 900 ms and a pitch of 1 were used. A total projection of 240 projects over 360° of rotation was acquired. Projection data were rebinned by 1:4 and reconstructed using a RamLak filter. Image files of PET and CT scan were fused using InVivoScope version 2.00. For quantitative tracer uptake analysis after the CT scan, the animals were perfused with PBS, the heart (divided into an ischemic and a non-ischemic part) and the quadriceps femoris (muscle control tissue) were removed and radioactivity was measured in the gamma counter (Perkin Elmer) using an energy window between 450 and 650 keV. Results are expressed as % injected dose per g (% ID/g) of tissue.

### Statistics

All quantitative data is reported as mean +/− standard deviation. Statistical analysis was performed using ANOVA followed by Tukey’s multiple comparison test, with *P* < 0.05 considered statistically significant.

## Additional Information

**How to cite this article**: Ziegler, M. *et al*. Highly Sensitive Detection of Minimal Cardiac Ischemia using Positron Emission Tomography Imaging of Activated Platelets. *Sci. Rep.* **6**, 38161; doi: 10.1038/srep38161 (2016).

**Publisher's note:** Springer Nature remains neutral with regard to jurisdictional claims in published maps and institutional affiliations.

## Supplementary Material

Supplementary Information

Supplementary Video S1
