# Supplementary material for: Highly Sensitive Detection of Minimal Cardiac Ischemia using Positron Emission Tomography Imaging of Activated Platelets
Source: Sci Rep. 2016 Dec 2;6:38161. doi: 10.1038/srep38161 (PMC5133579; doi:10.1038/srep38161)
Supplement: Supplementary Information [file srep38161-s1.pdf]

Supplementary Information

Highly Sensitive Detection of Minimal Cardiac Ischemia using Positron Emission Tomography Imaging of Activated Platelets

Melanie Ziegler[1,#], Karen Alt[1,2,#], Brett M. Paterson[3], Peter Kanellakis[4], Alex Bobik[4], Paul S. Donnelly[3], Christoph E. Hagemeyer[2,5,6,*], Karlheinz Peter[1,5,6,*,+]

Supplementary Video S1. 360° view of the PET/CT scan of the heart after 60 min occlusion and 2h post reperfusion.

Supplementary Note

We measured around 600 platelets / mg heart tissue using flow cytometry for the 10 min / 2 h pCI time point. The ischemic tissue of the heart weights around 50 mg. Therefore, we estimate to image a total number of 30,000 single platelets plus an additional number of activated platelets in platelet-aggregates and leukocyte-platelet-aggregates in the ischemic part of the heart for the mice that underwent 10 min of ischemia followed by 2 h reperfusion which we have not picked up in our flow cytometry gating strategy.
