# Supplementary figures and images for: Highly Sensitive Detection of Minimal Cardiac Ischemia using Positron Emission Tomography Imaging of Activated Platelets
Source: Sci Rep. 2016 Dec 2;6:38161. doi: 10.1038/srep38161 (PMC5133579; doi:10.1038/srep38161)

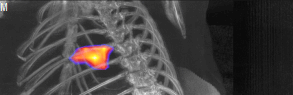

Supplement: Supplementary Video S1 [file srep38161-s2.gif]
